# Advanced Design and Fabrication of Dual-Material Honeycombs for Improved Stiffness and Resilience

**DOI:** 10.3390/mi14112120

**Published:** 2023-11-18

**Authors:** Jiajing Dong, Songtao Ying, Zhuohao Qiu, Xixi Bao, Chengyi Chu, Hao Chen, Jianjun Guo, Aihua Sun

**Affiliations:** 1 College of Mechanical Engineering, Zhejiang University of Technology, Hangzhou 310023, China; yingsongtao@nimte.ac.cn (S.Y.); qiuzhuohao@nimte.ac.cn (Z.Q.); baoxixi@nimte.ac.cn (X.B.); 2 Key Laboratory of Additive Manufacturing Materials of Zhejiang Province, Ningbo Institute of Materials Technology and Engineering, Chinese Academy of Sciences, Ningbo 315201, China; chucy@nimte.ac.cn (C.C.); chenhao21@nimte.ac.cn (H.C.); jjguo@nimte.ac.cn (J.G.)

**Keywords:** re-entrant honeycomb, finite-element simulation, dual-material printing, energy absorption

## Abstract

Auxetic re-entrant honeycomb (AREH) structures, consisting of a single soft or tough material, have long faced the challenge of balancing stiffness and rebound resilience. To achieve this balance, dual-material printing technology is employed to enhance shock absorption by combining layers of soft and tough materials. Additionally, a novel structure called the curved re-entrant honeycomb (CREH) structure has been introduced to improve stiffness. The selected materials for processing the composite structures of AREH and CREH are the rigid thermoplastic polymer polylactic acid (PLA) and the soft rubber material thermoplastic polyurethane (TPU), created utilizing fused deposition modeling (FDM) 3D printing technology. The influence of the material system and structure type on stress distribution and mechanical response was subsequently investigated. The results revealed that the dual-material printed structures demonstrated later entry into the densification phase compared to the single-material printed structures. Moreover, the soft material in the interlayer offered exceptional protection, thereby ensuring the overall integrity of the structure. These findings effectively serve as a reference for the design of dual-material re-entrant honeycombs.

## 1. Introduction

Honeycombs are widely utilized as biomaterials with distinctive periodic topological properties. These materials possess remarkable characteristics such as high fracture toughness, excellent impact resistance, as well as effective vibration and noise reduction. Meanwhile, honeycomb structures are reported to be better in mechanical performance compared to foams of the same density [1,2,3], and find extensive applications in various industries, including automotive, aerospace, and medical fields [4,5,6]. Among the various honeycomb structures used, auxetic re-entrant honeycombs (AREH) exhibit a distinct mechanical behavior when subjected to external forces due to their negative Poisson’s ratio [7]; these structures exhibit superior shear resistance and greater energy absorption efficiency [8,9]. When subjected to force or compression, they undergo lateral expansion or inward contraction, which is distinct from the mechanical behavior of traditional structures and is commonly referred to as metamaterial behavior [10]. Gibson et al. conducted extensive research on re-entrant honeycombs and proposed a formula for calculating their relative density [11]. Liu et al. [12] demonstrated that, under quasi-static compression, the re-entrant honeycomb structure absorbs more energy compared to the typical hexagonal honeycomb structure at the same degree of strain. Hu et al. [13] investigated the dynamic response of these structures utilizing the energy method and derived an analytical formula for the average crushing stress, considering the cell–wall angle, impact velocity, and cell–wall length ratio.

According to different loading directions, the AREH structure exhibits distinct energy absorption mechanisms. For example, under in-plane loading conditions, bending deformations of the cell walls and plastic hinges at the cell wall joints were found to be key to absorbing the energy [14]. During the compression process, the inclined walls of the cell undergo plastic deformation and squeeze each other. The specially designed negative Poisson’s ratio structure causes the entire honeycomb structure to collapse layer by layer in the plane, shrinking inward while absorbing a large amount of energy. This phenomenon is often seen in the stress–strain curve. When plastic deformation occurs in the honeycomb structure, the stress increases as the strain increases, [15] and the stress decreases rapidly after the failure of a layer of cells. Under out-of-plane loading conditions, the main modes of deformation are cell wall buckling and membrane deformations. However, the out-of-plane response does not fully reflect the negative Poisson’s ratio effect of the AREH structure. To enhance the distinctive mechanical properties of the structure, numerous researchers have explored various approaches, including modifying the cell structure or incorporating auxiliary structures [16,17,18,19]. Additionally, considerable research efforts have focused on developing improved optimization techniques to enhance specific aspects of the structure’s performance. Several existing optimization techniques include topology optimization [16,20,21], homogenization [22], and genetic algorithms [23].

Owing to the ligaments in the auxetic structure’s rotational or bending deformation properties, the stiffness of such structures is commonly not particularly high, making them unsuitable for situations that require high stiffness [24,25,26], energy absorption and strength [27]. However, the curved ligaments in natural structures such as turtle shells [28] provide some bionic ideas. These structures exhibit superior stiffness and enhanced energy absorption capabilities. Zied et al. [29] introduced two novel rigid plate auxetic honeycombs in which the straight walls were replaced with double-arc walls. This modification increases the in-plane stiffness while reducing auxeticity. The structure effectively dissipates impact energy over a larger area. Another honeycomb arrangement called the re-entrant circle (REC), developed by Chang et al. [17], replaces the inclined honeycomb walls with double-circular-arc honeycomb walls. The additional plastic hinges in this configuration increase the energy required for each unit during the crushing process, substantially enhancing the structure’s energy absorption capacity. Meanwhile, it has been demonstrated that the multilayered hierarchical structure found in avian beaks [30] and nacre [31] can be employed to disperse and absorb impact energy. Niu et al.’s [32] study on the integrated production of soft and hard composite materials of various sizes under various circumstances led to the realization of the controllability and mechanical property enhancement of tunable soft and hard hybrid structures. By using softer polymers in the hinge area, Wang et al. [33] suggested that 3D re-entrant honeycombs could utilize dual-material printing to reduce buckling issues. To enhance the functionality of chiral auxiliary metamaterials, Zhang et al. [34] proposed a gradient-based mathematical programming technique employing topology optimization. Su et al. innovatively introduced the concept of dual-material printing and incorporated a soft arch ligament into the re-entrant honeycomb, which improved stiffness while retaining the NPR characteristics. Similarly, ROSS et al. [35] developed a series of dual-material combinations, similar to Su et al. [36], using soft connections at the hinges. Experimental results revealed that the dual-material structure exhibited excellent rebound resilience under multiple loading cycles and compressive forces. Previous research had incorporated soft materials into the honeycomb structure’s hinges to increase rebound resilience, but this approach compromised the stiffness.

In this study, dual-material printing was incorporated to achieve a better balance between bounce resilience and energy absorption capacity. The samples were created via an FDM printing technique using two different types of materials. The deformation mode of these new structures with various materials and configurations was predicted and evaluated using a quasi-static test, integrating finite-element (FEM) simulation and experiments. The experimental findings were presented, comparing the stiffness and specific energy absorption (SEA) of the newly developed re-entrant honeycomb structures to conventional honeycomb structures. Additionally, the experimental results for the dual-material structure were compared to those obtained from a single material. These findings provide valuable insights for dual-material printing and serve as reference data for printing dual-material honeycomb structures using FDM printing technology.

## 2. Materials and Methods

### 2.1. Fabrication and Mechanical Properties Characterization

**Figure 1:**
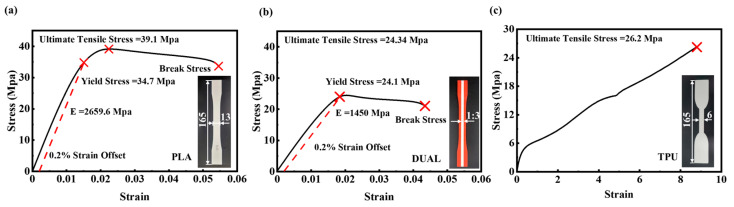
Stress–strain curve for the (**a**) PLA material (**b**) DUAL material and (**c**) TPU material.

The tested samples were fabricated via fused deposition modeling (FDM) technology, using PLA filament (Raise3D, Shanghai, China) and TPU95A (PolyFlex, Brisbane, Australia) as raw materials. These materials were selected due to their different stiffness characteristics. The detailed print parameters can be found in Table S1 (Supplementary Materials). Moreover, the tensile test was utilized to characteristic the elastic modulus and yield strength of the samples. For hard plastics, the standard tested methods of PLA samples followed ASTM D638-I [36,37], and TPU samples were fabricated following ASTM D638-IV [20,38]. Meanwhile, the dual-material samples were made following ASTM D638-I, with the middle slender consisted with PLA and TPU [39]. Then, their stress–strain curves were measured and displayed in Figure 1a–c.

### 2.2. Structure and Material Designs of AREH and CREH

**Figure 2:**
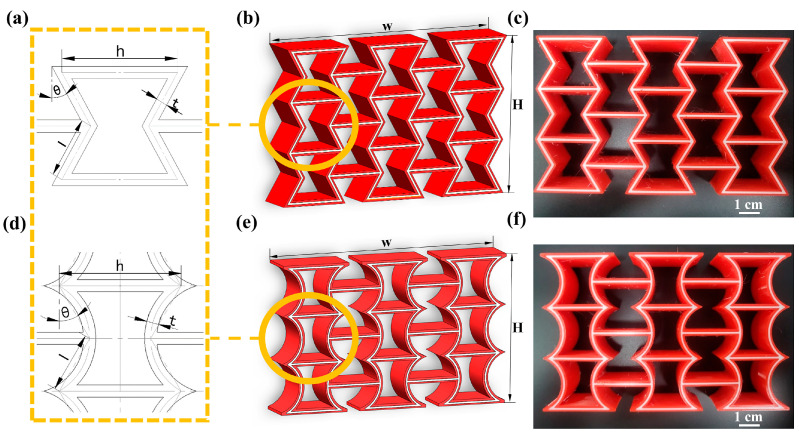
Schematic diagram of honeycomb cell and sandwich composite structure and manufactured specimen of (**a**–**c**): AREH, (**d**–**f**): CREH.

The geometric shape of the unit cell in AREH structure is usually determined by four parameters of its basic unit, as in shown in Figure 2a, which are the length of the inclined rod (l), the length of the vertical rod (h), the wall thickness (t) and the concave angle (θ). The length of the rod on the left and right sides is 1/2 h; all rod elements have rectangular sections. At the same time, to ensure that the structure exhibits a negative Poisson’s ratio effect during deformation, these parameters must satisfy 2lsinθ < h. In order to avoid the overlap between the inclined rod and the vertical rod, it is necessary to ensure that lcosθ > t. In this study, the specific sizes were l = 15 mm, h = 30 mm, t = 3 mm, and θ = 30°. The overall structural dimensions obtained by the array were w = 125 mm and H = 80 mm, and the thickness of the whole structure was 20 mm. There are 6 nodes in a single AREH cell, which are symmetrically distributed. There are 3 nodes on one side. These 3 nodes were drawn into arcs and arranged symmetrically. This new structure with arched walls was named curved re-entrant honeycomb (CREH), as shown in Figure 2d.

To imitate the soft and hard sandwich structure seen in biology [30,31], the dual material design of these two structures was continued. The whole structure was divided into two parts from the wall thickness direction as shown in Figure 2b,e. The internal design is a TPU sandwich layer with a wall thickness of 1 mm, and the external design is a two-layer PLA shell with a thickness of 1 mm. It is fabricated using dual-material printing technology, as shown in Figure 2c,f.

### 2.3. Finite-Element Method Simulation

**Figure 3:**
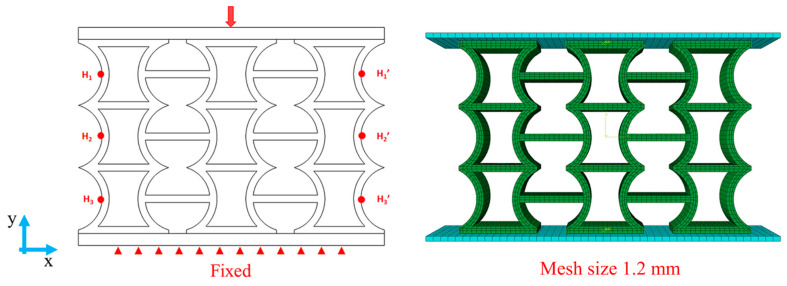
Schematic diagram of finite-element method simulation.

Finite-element modelling was processed via the Abaqus/CAE package. The explicit method was chosen [40]. Models for these structures were created via SolidWorks 2019 and imported in IGES format. C3D8R-type mesh components were utilized for meshing. The material properties of PLA and TPU were determined through a fitting process, as is shown in Table S2. Two rigid plates were fixed at the top and bottom and a reference point was established at the center of the top rigid plate for stress and displacement measurements. To simulate the quasi-static compression experiment [16], a displacement of 45 mm was applied to the top rigid body plate along the -Y direction. Figure 3 illustrates the setup of the model between the two rigid plates. To define contact behavior between surfaces, a tangential behavior penalty coefficient of 0.2 was used as the interaction attribute [16,35]. After mesh division, a mesh interface was established for the compressed sample to establish the general contact between surfaces and achieve self-contact functionality.

For the dual-material printed structures, the exterior and sandwich structures were imported separately, and the merging function was employed to combine the two sections for computation. It should be noted that each section had its own set of material attributes.

Figure S2 provides a mesh sensitivity analysis, and a mesh size = 1.2 mm was selected. Meanwhile, Poisson’s ratio was calculated by selecting two reference points, denoted as $H_{i}$ and $H_{i}^{\prime }$, with equal intervals on the left and right sides of the boundary [41]. The specific calculation method can be obtained in [41]. The specific calculation method is described by Equations (S4)–(S7).

## 3. Results and Discussion

### 3.1. Analysis Methods

**Figure 4:**
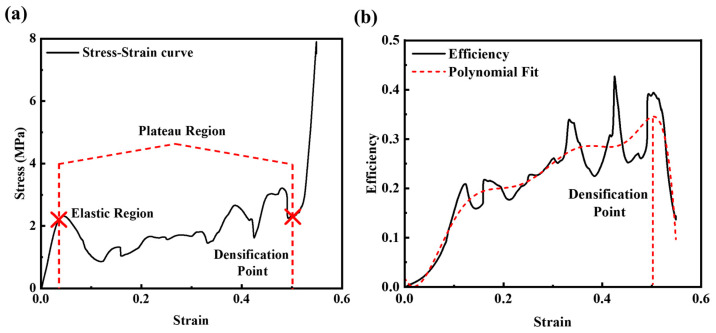
(**a**) The stress–strain curve and (**b**) the energy efficiency curve of AREH structure printed with PLA material.

In this study, the quasi-static compression experiment and finite-element simulation are mainly carried out for the two printing methods to obtain the stress-strain curve, and the Poisson’s ratio is extracted to analyze the auxetic characteristics. The quasi-static compression test can be followed after printing the samples so that the stress–strain curve can be obtained. In Figure 4a, it can be clearly seen that there are three stages, namely, the elastic stage, plateau stage and densification stage. In the elastic stage, the image shows an upward trend with a constant slope, which is Young’s modulus. The stress reduction at the yield point is due to the rotation of the plastic hinge and the bending of the cell walls, after which the structure enters the stress plateau region. In this process, the collapse is accompanied by a near constant load, creating a stress plateau. The third stage is the densification stage: the collapsed cell walls are squeezed tightly against each other, causing the stress to rise sharply and indicating that the structure has failed.

Li et al. [42] proposed a method to evaluate the densification of a structure by calculating its energy absorption efficiency during compression. This method was used to analyze the energy absorption efficiency of the AREH structure, as shown in Figure 4b. Additionally, this method can be applied to various structures made of different materials. The detailed procedure and content of energy absorption can be calculated by Equations (S8)–(S10).

### 3.2. Experiments and FEM Simulations

The finite-element model (FEM) of the specimen was established, and the quasi-static experiment was simulated. Table 1 presents the deformation of AREH samples printed with either a single TPU or PLA material during the compression crushing process, along with the predicted deformation mode profile obtained from simultaneous finite-element simulations.

#### 3.2.1. Deformation Mode of the AREH Samples

The TPU-AREH sample demonstrated exceptional flexibility and remained undamaged throughout the entire loading process. When pressed into 20 mm, the structure inclined to one side and then expanded outward rapidly, which was caused by the remarkable elasticity of TPU. This resulted in visible and significant deformation of the entire structure, quickly transitioning into a densification stage.

**Figure 5:**
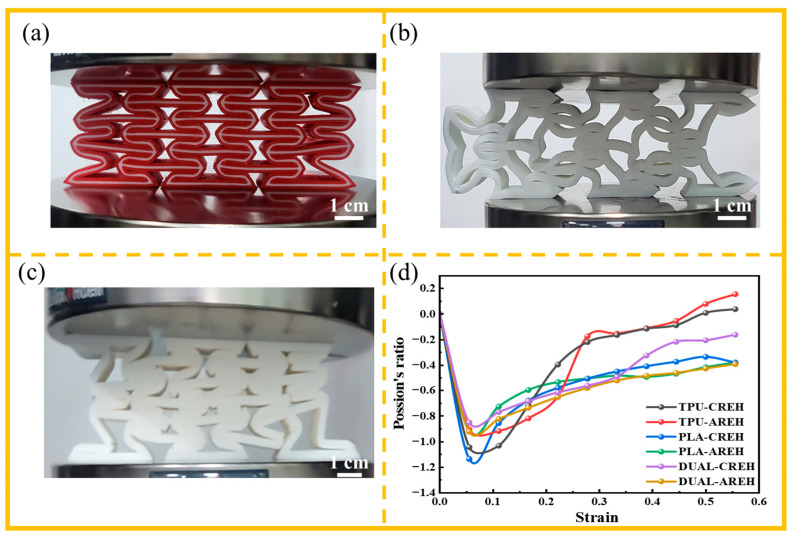
Comparison of compression testing in AREH structures using different materials: (**a**) DUAL (**b**) TPU (**c**) PLA and (**d**) Poisson’s ratio of all samples.

**Figure 6:**
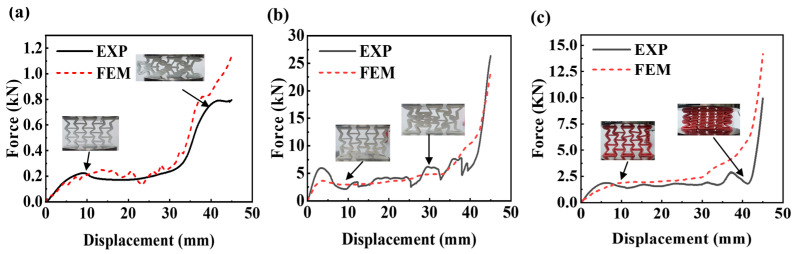
Comparison of Force—displacement Curves of the AREH structure between simulation and experiment: (**a**) TPU (**b**) PLA (**c**) DUAL.

Figure 5d illustrates the instantaneous Poisson’s ratio of each structure during the compression process, which was obtained using the FEM simulation described in Section 2.3. It can be observed that the Poisson’s ratio of each sample decreases at different rates but reaches its lowest value at a similar strain value before subsequently increasing. By comparing the AREH samples with different materials listed in Table 1, it is evident that the PLA-AREH sample primarily exhibits elastic deformation during the initial loading stage. The structure then enters plastic deformation, which is manifested by the rotation of the plastic hinge and the bending of the wall. This is shown in Figure 6b as a rapid decrease in force. This is shown in Figure 5d, where the Poisson’s ratio of the structure rapidly decreases to −0.96. Subsequently, obvious stress concentration occurs at the plastic hinge, and it breaks quickly after torsion. In Figure 5c, it can be observed that the central cell is completely crushed, leading to layer-by-layer fracturing and inward shrinking throughout the entire structure.

The FEM simulation indicates significant stress concentration in the cells located along the diagonal line within the overall structure. Experimental results confirm severe deformation and even fracturing in these regions. The cell located at the center experiences the most extensive damage, resulting in inward contraction. This specific deformation pattern causes individual cells to gradually move towards the center of the specimen along the diagonal direction, generating a significant negative Poisson’s ratio effect. However, a lower Poisson’s ratio leads to earlier densification, as evident in Figure 5b, where the TPU-AREH structure exhibits the lowest Poisson’s ratio among the AREH structures, with a value of −1.09. In comparison, Figure 5a demonstrates that the DUAL-AREH structure exhibits better structural integrity, enters the densification stage later, and shows no cell fractures or damage.

In contrast to pure TPU or PLA samples, the plastic hinges and cell walls of the DUAL-AREH sample exhibited more stable rotation and deformation, allowing them to withstand compression loads without breaking. This dual-material printed AREH sample presented the unique properties of the dual-material layering printing method, which includes layer-by-layer compaction and enhanced stiffness. Figure 5a indicated that the cell walls of the DUAL-AREH printed sample do not experience wall fracturing or shedding of the dual-material layers and inter-layers throughout the entire crushing process.

#### 3.2.2. Deformation Mode of the CREH Samples

**Figure 7:**
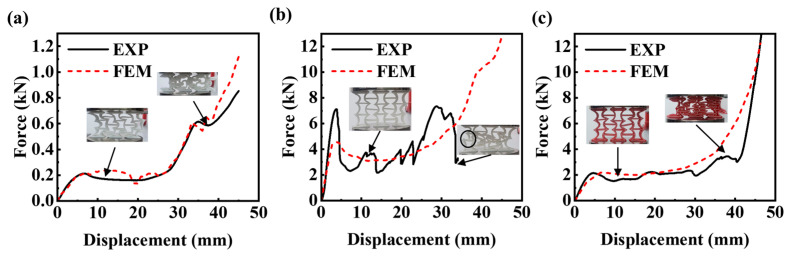
Comparison of force—displacement curves of the CREH structure between simulation and experiment: (**a**) TPU (**b**) PLA (**c**) DUAL.

The inclined wall of the re-entrant honeycomb was converted into an arc wall, and the pure PLA and TPU samples were compressed quasi-statically. FEM simulation described the stress distribution of the structure during compression and the force–displacement diagram obtained via experimentation and FEM simulation fit well, as is shown in Figure 7. In Table 2. The deformation mode of the CREH structure printed with TPU was similar to that of the TPU-AREH structure. After loading, with the rotation of the plastic hinge and the bending of the wall, the cell in the middle quickly protruded to one side after a brief inward contraction.

CREH samples printed using PLA differ significantly from AREH samples. The arc-shaped wall has higher stiffness but different deformation forms than the AREH structure during quasi-static compression. PLA is brittle at room temperature, and the arch ligament further accelerates the fracturing caused by stress concentration, as predicted in the FEM simulation. During the experiment, it was found that the PLA-CREH structure was accompanied by oblique collapse and instantaneous large displacement when it failed, so that there was a large fluctuation in the force–displacement curve. After testing seven samples, it was confirmed that the structure had a shedding of the cell wall (Figure 7b) in the middle part when it was pressed into 28–33 mm. This structural damage would lead to premature failure compared to the AREH structure.

When the dual-material printed CREH samples were compressed to a depth of 16 mm, one side of the interface exhibited detachment. During compression, longitudinal failure is closely linked to and controlled by the in-plane shear response of the composite [43]. The inhomogeneous dissipation of stress on the dual-material joint surface results in the peeling of the PLA and TPU bonding layers [44]. As a result, a few TPU layers were pulled out from the structure due to their high fracture elongation, and some energy was consumed through friction. The overall deformation trends of the dual-material printed samples remains similar to those of TPU printed samples. Therefore, it can be inferred that the deformation mode of the CREH structure is primarily governed by the deformation of the TPU material. While this deformation mode still exhibits negative Poisson’s ratio characteristics, adjustments to the structural design are necessary in practical applications to prevent interference with other structures.

**Figure 8:**
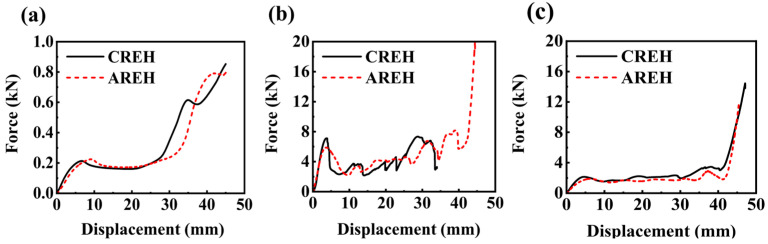
Comparison of force–displacement curves for samples of different structures printed with different materials: (**a**) TPU (**b**) PLA (**c**) DUAL.

Table 3 presents the elastic modulus of structures printed with different materials. Based on the quasi-static compression curves depicted in Figure 8, which illustrate samples with the same material but different structures, it can be observed that the elastic modulus of CREH configuration samples printed with TPU, PLA, and DUAL materials increased by 47.15%, 24.68% and 53.86%, respectively, compared to the AREH configuration. This indicates that the CREH configuration is better suited for applications requiring higher stiffness.

Furthermore, the yield stress of the AREH structures printed with PLA and dual materials increased by 20.85% and 13.33%, respectively, from 2.35 MPa and 0.75 MPa to 2.84 MPa and 0.85 MPa. However, the CREH structure constructed using TPU material did not show advantages, which was due to the low stiffness of the hyperelastic material itself and the similar deformation modes of the two structures.

### 3.3. Advantages of the Dual-Material Structures

**Figure 9:**
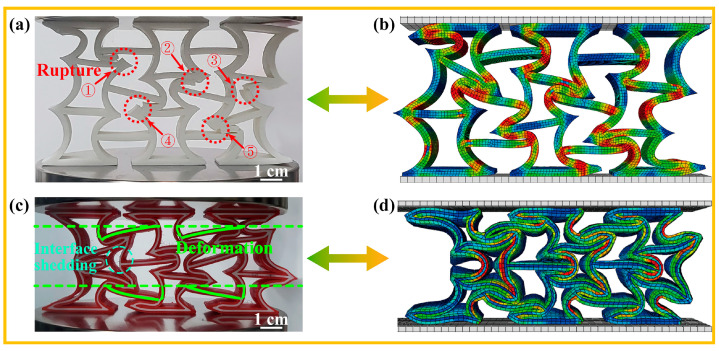
Details in the quasi-static compression process compared with FEM: (**a**) EXP-PLA (**b**) FEM-PLA (**c**) EXP-DUAL (**d**) FEM-DUAL.

When reinforced by TPU interlayers, dual-material printed CREH samples demonstrate excellent flexibility, as is shown in Figure 9c. Meanwhile, the TPU intermediate rubber absorbs shock and energy, preventing stress concentration from plastic hinges and delaying the onset of further damage. Due to the higher stiffness of the CREH structure, there will be visible stress concentrations following wall bending. This is shown in the wall bending and breaking damage in the PLA printed samples, as well as the detachment between interfaces in the dual-material printed samples. When the samples printed with the CREH structure enter the stress plateau region, Figure 5d shows a rapid decrease in the Poisson’s ratio of the PLA-CREH structure, reaching a minimum value of −1.17, these outcomes cause earlier densification stage [35].

**Figure 10:**
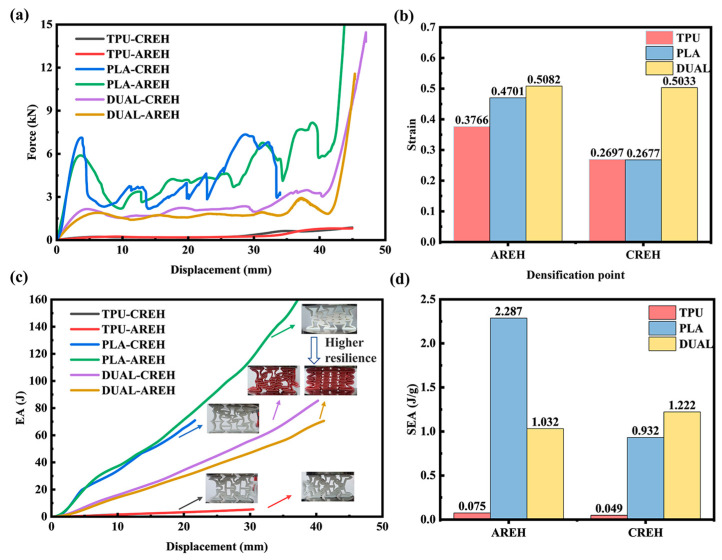
Performance comparison of all samples: (**a**) force–displacement curves, (**b**) densification point, (**c**) total energy absorption (EA) up to densification stage (**d**) SEA.

The densification points and absorbed energy of the samples were calculated using Equations (S4) and (S5), as depicted in Figure 10b,c. Dual-material printing technology effectively delays the densification stage of the honeycomb structure in both the AREH and CREH configurations. When compared to pure TPU and PLA samples, the densification strain of the DUAL-AREH structure increases by 34.94% and 8.10%, respectively. This increase is even more pronounced in the CREH configuration, with respective increments of 86.61% and 88.0%. This is because the CREH structures possess higher stiffness, as shown in Figure 10a. During the compression process, a large number of stress concentration points are generated due to the rotation of the plastic hinge, leading to ruptures in these parts (From 1 to 5 in Figure 9a). By appropriately incorporating a soft–hard composite, the densification process can be delayed, thus preventing premature structural failure. However, the densification strain of the DUAL-CREH structure is very close to that of the DUAL-AREH structure, which can be caused by the delamination of the soft and hard interfaces of the CREH structure, leading to earlier densification of the DUAL-CREH structure.

In Section 2.2, where the central part of the structures was removed to print the soft materials. This design sacrificed structural integrity and reduced the specific energy absorption (SEA). As shown in Figure 10d, the SEA of the DUAL-AREH sample was 1.032 J/g, which was approximately half of that calculated in the PLA-AREH structure (2.287 J/g). However, the DUAL-CREH structure exhibited a noteworthy increase in SEA compared to PLA-CREH sample, changing from 0.932 J/g to 1.222 J/g; this represented a 31.12% improvement. This can be attributed to the fact that DUAL-CREH structures printed with dual materials do not fail prematurely and have later densification, leading to more energy absorption.

**Figure 11:**
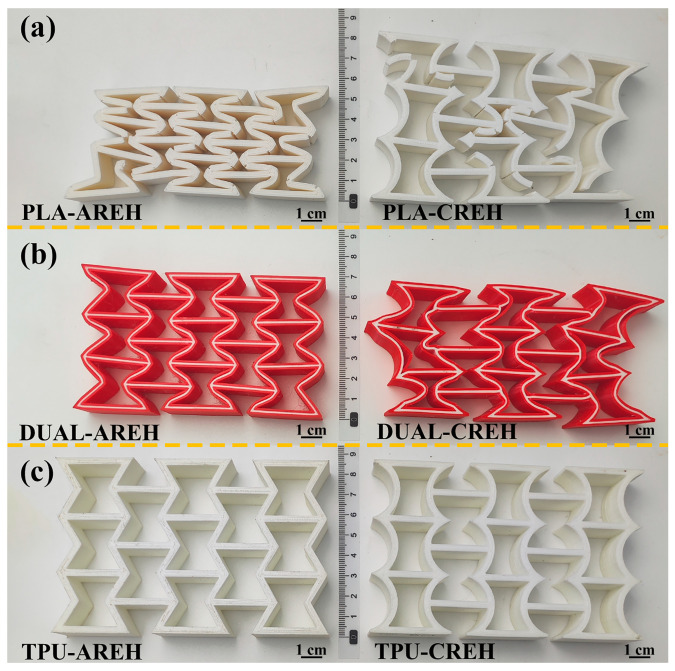
Samples after releasing the load: (**a**) PLA, (**b**) DUAL, (**c**) TPU.

Meanwhile, dual-material printed samples clearly exhibit rebound resilience after compression testing. As shown in Figure 11, all the DUAL samples experienced a significant rebound from 36 mm to almost 63 mm in the minimal spring back area. It was observed that the height of the pure TPU samples rapidly rebounded to 84% of the original height after the load was removed and returned to 100% of the original size after standing for 1 h. This rebound behavior is determined by the material properties of TPU. However, such rebound characteristics are not observed in the PLA material. The experimental results demonstrate that the DUAL samples, printed using both TPU and PLA materials, obtain a certain resilience of 33.3% from the TPU layer present in the interlayer.

## 4. Conclusions

In this work, a unique curved re-entrant honeycomb (CREH) structure was designed based on auxetic re-entrant honeycomb (AREH) structures. Various composite structures were fabricated using the dual-material fused deposition modeling (FDM) technique, combining soft and rigid layers. Combined with quasi-static compression tests, the mechanical performance and deformation modes of the composite structures were analyzed using finite-element simulation methods under different material combinations. The results indicate that the CREH structure exhibits higher stiffness compared to the AREH structure; the elastic modulus of the CREH samples printed with TPU, PLA, and dual materials increased by 47.15%, 24.68%, and 53.86%, respectively. In addition, the incorporation of the soft TPU material effectively alleviates stress concentration issues and prevents rapid densification or failure via rupture, particularly for the CREH structures which possess higher rigidity. The densification strain of the dual-material printed CREH structure increased by 86.61% and 88.0% compared to the use of single TPU and PLA materials, respectively. In addition, the dual-material printed samples also exhibited a resilience not possessed by PLA, reaching 33.33%. This capability is attributed to the soft interlayer, which enables the structure to withstand multiple loads. Dual-material printing provides more design inspiration for complex lightweight energy-absorbing honeycomb structures, particularly for materials prone to brittle fracturing, such as polylactic acid (PLA). By altering the filling ratio, filling structure, and incorporating various material combinations, including suitable mechanical interlocking structures, the requirements of different working conditions can be met.

## Figures and Tables

**Table 1:** The sequence of deformed configurations during deformation and failure of the AREH samples.

mm	TPU-AREH	FEM	PLA-AREH	FEM	DUAL-AREH	FEM
10	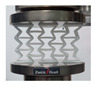	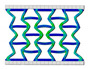	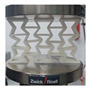	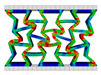	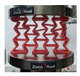	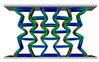
20	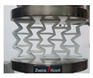	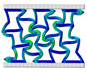	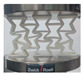	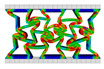	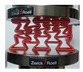	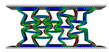
30	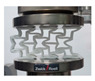	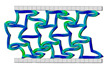	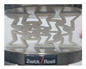	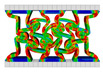	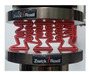	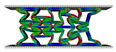
40	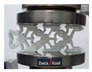	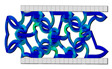	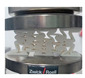	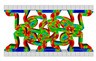	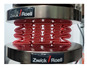	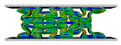

**Table 2:** The sequence of deformed configurations during deformation and failure of the CREH samples.

mm	TPU-CREH	FEM	PLA-CREH	FEM	DUAL-CREH	FEM
10	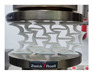	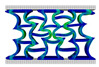	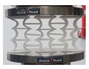	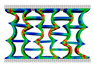	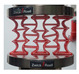	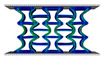
20	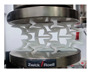	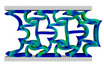	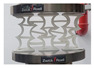	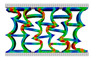	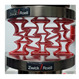	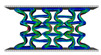
30	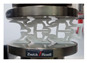	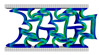	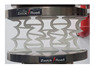	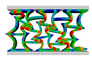	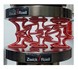	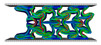
40	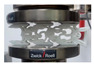	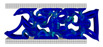	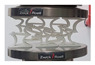	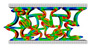	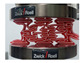	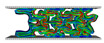

**Table 3:** The mechanical performance parameters of AREH and CREH samples.

	Elastic Modulus (MPa)	Yield Stress (MPa)	Energy Absorption Efficiency (Max)	Energy Absorption (J)
TPU-AREH	1.23	0.07	0.27	5.34
TPU-CREH	1.81	0.05	0.25	3.73
PLA-AREH	68.14	2.35	0.33	166.47
PLA-CREH	84.96	2.84	0.25	68.45
DUAL-AREH	15.69	0.75	0.39	75.22
DUAL-CREH	24.14	0.85	0.36	85.69

## Data Availability

Data are contained within the article and Supplementary Materials.

## Supplementary Materials

The following supporting information can be downloaded at: https://www.mdpi.com/article/10.3390/mi14112120/s1, Figure S1. Zwick/Roell Z100 Universal test machine. Figure S2. Mesh sensitivity analysis. Table S1. Print parameters of the tensile tested specimen. Table S2. Polynomial data to curve fitting the TPU. Refs. [45,46] are cited in the Supplementary Materials.
