# Supplementary material for: Advanced Design and Fabrication of Dual-Material Honeycombs for Improved Stiffness and Resilience
Source: Micromachines (Basel). 2023 Nov 18;14(11):2120. doi: 10.3390/mi14112120 (PMC10673381; doi:10.3390/mi14112120)
Supplement: Supplementary file 1 [file micromachines-14-02120-s001.zip › micromachines-2696411-supplementary.pdf]

Supplementary material

Advanced design and fabrication of dual-material honeycombs for improved stiffness and resilience

Jiajing Dong[ab]*, *SongtaoYing*[ab], *Zhuohao Qiu*[ab], *Xixi Bao*[ab], *Chengyi Chu*[b], *Hao Chen*[b], *Jianjun Guo*[b], *Aihua Sun*[b]*

[a]*College of Mechanical Engineering, Zhejiang University of Technology, Hangzhou 310023, China*

[b] *Key Laboratory of Additive Manufacturing Materials of Zhejiang Province, Ningbo Institute of Materials Technology and Engineering, Chinese Academy of Science, Ningbo 315201, PR China*

Corresponding author:

Jiajing dong

E-mail address: dongjiajing@nimte.ac.cn

Aihua sun

E-mail address: sunaihua@nimte.ac.cn

Tensile test and spline data processing

As illustrated in Figure S1, a Zwick/Roell Z100 Universal test apparatus was used to test four specimens of each material, with displacement rates of 4 mm/min for PLA[1] and 10 mm/min for TPU[2]. The average stress-strain graphs for these testing are given in Figure 1, which shows that the samples were discovered to be reproducible. The PLA displays an initial linear-elastic phase, which makes it possible to determine Young's modulus (E). The stress equivalent to 0.2% plastic strain is typically used as the yield strength or yield limit for plastic materials lacking an evident yield stage[3]. The Young's modulus of TPU, which is a hyperelastic material that is difficult to measure directly and has a failure strain of up to 840%. The tensile experiment provides the engineering stress and strain, but the finite element analysis program requires the actual stress and strain, which may be obtained by applying Eq. S1 and S2 to the instantaneous cross-sectional area of the tensile experiment[3].

$$\mathrm{\sigma_t = \sigma_e(1 + \varepsilon_e)} \tag{S1}$$

$$\varepsilon_t = ln\,(\varepsilon_e + 1) \tag{S2}$$

In which σ_t is true stress, σ_e is engineering strain and ε_e is true strain.

To calculate the plastic strain for the PLA, Eq. S3 was used[3].

$$\varepsilon_{plastic} = \varepsilon_t - \frac{\sigma_t}{E} \tag{S3}$$

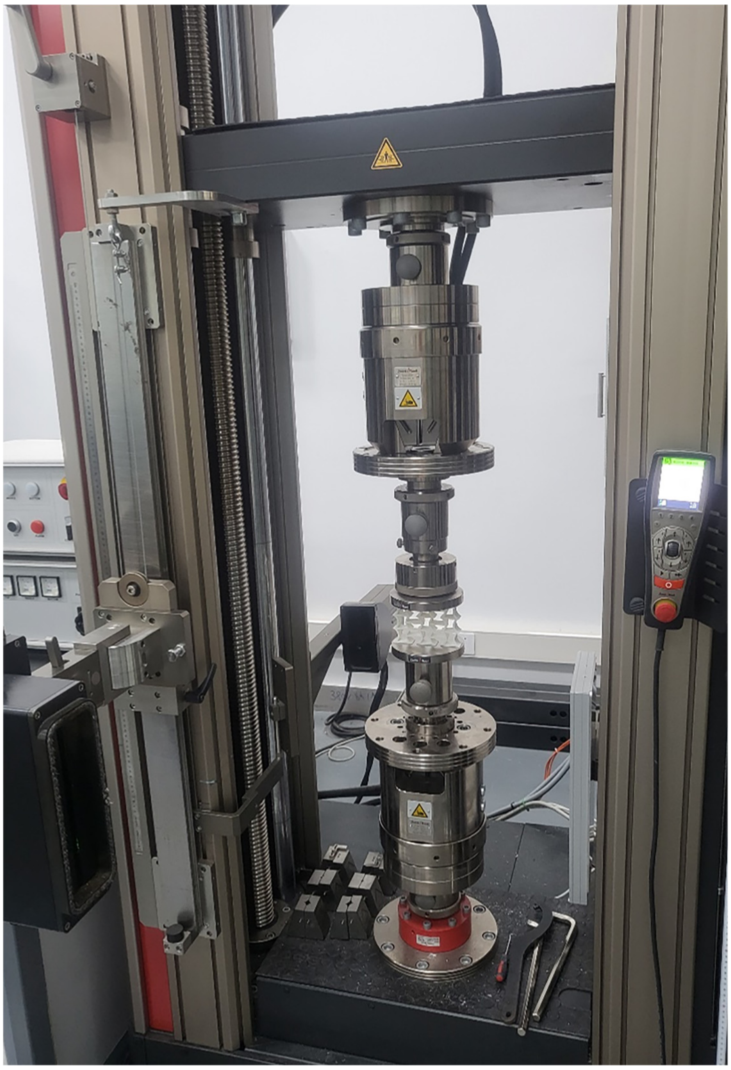

Figure S1. Zwick/Roell Z100 Universal test machine.

Table S1

Print parameters of the tensile tested specimen.

	Nozzle diameter (mm)	Layer Height (mm)	Printing speed (mm/s)	Fill density (%)	Nozzle temperature (^{o}C))	Bed temperature (^{o}C)
PLA	0.4	0.2	60	100	205	55
TPU	0.4	0.2	35	100	225	55

Material property fitting

In this study, PLA and TPU were used as the materials, and the material selection was determined by the input of material characteristics collected from the prior experimental data. TPU requires further curve fitting. Enter the TPU curve data from Figure 1.

To choose the appropriate hyperplastic criterion. In this model, we employed the Abaqus analysis module to fit the hyperplastic material, and a quadratic polynomial in line with the experimental data was derived, with the results displayed in Table.S2. The constitutive model was then transformed into a quadratic polynomial and entered each value.

Table S2

Polynomial data to curve fitting the TPU.

D1	C10	C01	C02
D2	C20	C11	
0	-8.4954	14.1790	2.6702
0	2.801E-03	-4.65E-02	

Mesh sensitivity analysis

Mesh sensitivity was performed by changing the approximate global size of elements. As it is shown in Figure S2 by changing the mesh size, the results remain steady for the elements whose mesh size were 1.2 mm or less. Therefore, to meet the accuracy and reduce the computation time, the mesh size 1.2 mm were chosen.

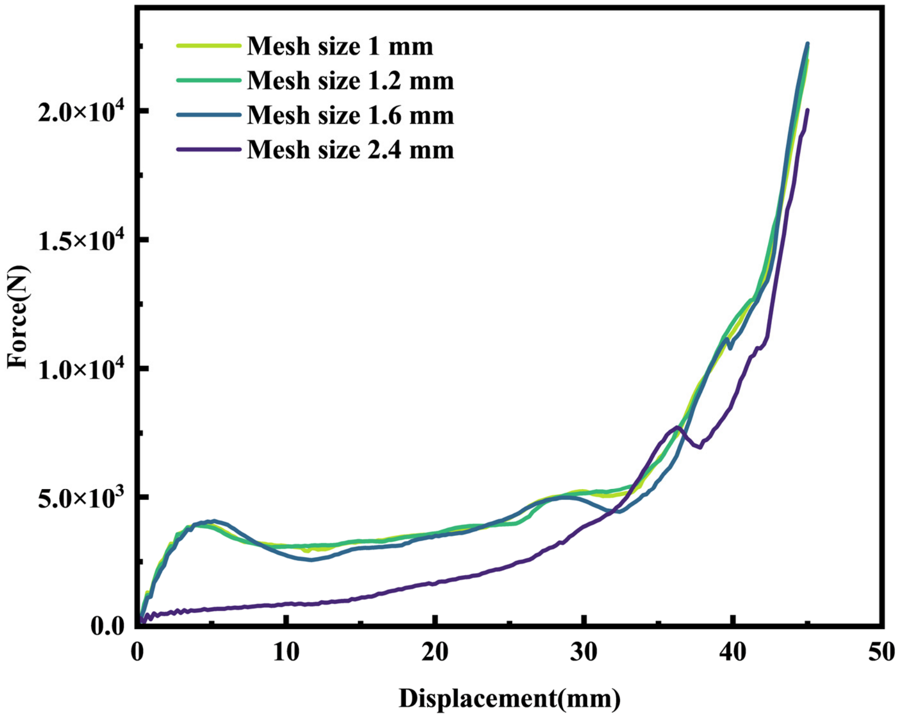

Figure S2. Mesh sensitivity analysis.

Poisson's ratio for the honeycomb structure at a specific strain is determined by calculating the average value of Poisson's ratio [2] using pairs of reference points. The calculation method for Poisson's ratio is described by Equation (S4–S7):

$$\varepsilon_{x_i} = (H_0H_0' - H_iH_i')/H_0H_0' \quad \text{(S4)}$$

$$\varepsilon_{yi} = \varepsilon_{nominal\ strain} \quad \text{(S5)}$$

$$\nu_i = -\frac{\varepsilon x_i}{\varepsilon y\mathrm{i}} \quad \text{(S6)}$$

$$\nu = \frac{\sum_{i=1}^{3} \nu_i}{3} \quad \text{(S7)}$$

Where ε_{x_i} is horizontal strain, ε_{yi} is transverse strain, H_0H_0' is 108 mm, the Poisson 's ratio of the honeycomb is the average value of the Poisson 's ratio calculated by each pair of data.

The specific method and content of energy absorption

The advantage of this method by calculating the energy absorption efficiency as

shown in Figure 4b, find the peak of the curve, namely called densification point, which is the strain value at the end of the stress plateau area as shown in the Figure 4a. A quintic polynomial is very important to smooth the energy absorption efficiency curve in order to obtain the densification point because we discover that the noise in the calculating process is obvious. This energy absorption efficiency is defined by

$$\eta(\varepsilon) = \frac{1}{\sigma(\varepsilon)} \int_0^{\varepsilon} \sigma(\varepsilon)\, d\varepsilon \qquad \text{(S8)}$$

Where ε is the strain, $\sigma(\varepsilon)$ is the stress corresponding to the strain. After the densification point is obtained, the strain at which it is located is converted into displacement, and the position is found in the force-displacement image. The absorbed energy value can be obtained by the definite integral of force-displacement [4], and the absorbed energy is the work done by the internal structure of the structure under compression.

$$W = \int_0^{\delta} F\, dx \qquad \text{(S9)}$$

Where δ is the densification point. In the evaluation of energy absorption characteristics, specific energy absorption (SEA) is used to conduct the study, which represents the energy absorbed per unit mass (m).

$$SEA = \frac{w}{m} \qquad \text{(S10)}$$

References

[1] C. Qi, F. Jiang, C. Yu, S. Yang, In-plane crushing response of tetra-chiral honeycombs, International Journal of Impact Engineering 130 (2019) 247-265.http://doi.org/10.1016/j.ijimpeng.2019.04.019

[2] R. Hamzehei, J. Kadkhodapour, A.P. Anaraki, S. Rezaei, S. Dariushi, A.M. Rezadoust, Octagonal auxetic metamaterials with hyperelastic properties for large compressive deformation, International Journal of Mechanical Sciences 145 (2018) 96-105.http://doi.org/10.1016/j.ijmecsci.2018.06.040

[3] R. Johnston, Z. Kazancı, Analysis of additively manufactured (3D printed) dual-material auxetic structures under compression, Additive Manufacturing 38 (2021).http://doi.org/10.1016/j.addma.2020.101783

[4] D. Sharma, S.S. Hiremath, Experimental and FEM study on the in-plane and out-plane loaded reversible dual-material bio-inspired lattice structures with improved energy absorption performance, Composite Structures 303 (2023).http://doi.org/10.1016/j.compstruct.2022.116353
